# Supplementary material for: Bayesian refinement of protein structures and ensembles against SAXS data using molecular dynamics
Source: PLoS Comput Biol. 2017 Oct 18;13(10):e1005800. doi: 10.1371/journal.pcbi.1005800 (PMC5662244; doi:10.1371/journal.pcbi.1005800)
Supplement: S1 Archive — (BZ2) [file pcbi.1005800.s011.tar.bz2 › gromacs_4.62_bayesian_saxs_refi/share/html/online/g_helixorient.html]

g\_helixorient


| g\_helixorient Main Table of Contents |

**VERSION 4.6  
Sat 19 Jan 2013**

---

### Description

g\_helixorient calculates the coordinates and direction of the average
axis inside an alpha helix, and the direction/vectors of both the
Cα and (optionally) a sidechain atom relative to the axis.

As input, you need to specify an index group with Cα atoms
corresponding to an α-helix of continuous residues. Sidechain
directions require a second index group of the same size, containing
the heavy atom in each residue that should represent the sidechain.

**Note** that this program does not do any fitting of structures.

We need four Cα coordinates to define the local direction of the helix
axis.

The tilt/rotation is calculated from Euler rotations, where we define
the helix axis as the local x-axis, the residues/Cα vector as y, and the
z-axis from their cross product. We use the Euler Y-Z-X rotation, meaning
we first tilt the helix axis (1) around and (2) orthogonal to the residues
vector, and finally apply the (3) rotation around it. For debugging or other
purposes, we also write out the actual Euler rotation angles as theta[1-3].xvg

### Files

| option | filename | type | description |
| --- | --- | --- | --- |
| **-s** | topol.tpr | Input | Run input file: tpr tpb tpa |
| **-f** | traj.xtc | Input | Trajectory: xtc trr trj gro g96 pdb cpt |
| **-n** | index.ndx | Input, Opt. | Index file |
| **-oaxis** | helixaxis.dat | Output | Generic data file |
| **-ocenter** | center.dat | Output | Generic data file |
| **-orise** | rise.xvg | Output | xvgr/xmgr file |
| **-oradius** | radius.xvg | Output | xvgr/xmgr file |
| **-otwist** | twist.xvg | Output | xvgr/xmgr file |
| **-obending** | bending.xvg | Output | xvgr/xmgr file |
| **-otilt** | tilt.xvg | Output | xvgr/xmgr file |
| **-orot** | rotation.xvg | Output | xvgr/xmgr file |

### Other options

| option | type | default | description |
| --- | --- | --- | --- |
| **-[no]h** | bool | no | Print help info and quit || **-[no]version** | bool | no | Print version info and quit || **-nice** | int | 19 | Set the nicelevel || **-b** | time | 0 | First frame (ps) to read from trajectory || **-e** | time | 0 | Last frame (ps) to read from trajectory || **-dt** | time | 0 | Only use frame when t MOD dt = first time (ps) || **-xvg** | enum | xmgrace | xvg plot formatting: xmgrace, xmgr or none || **-[no]sidechain** | bool | no | Calculate sidechain directions relative to helix axis too. || **-[no]incremental** | bool | no | Calculate incremental rather than total rotation/tilt. |

---

http://www.gromacs.org  
gromacs@gromacs.org
